# Carbon Nanofiber Cement Sensors to Detect Strain and Damage of Concrete Specimens Under Compression

**DOI:** 10.3390/nano7120413

**Published:** 2017-11-24

**Authors:** Oscar Galao, F. Javier Baeza, Emilio Zornoza, Pedro Garcés

**Affiliations:** Department of Civil Engineering, University of Alicante, 03690 San Vicente del Raspeig, Spain; oscar.galao@ua.es (O.G.); fj.baeza@ua.es (F.J.B.); emilio.zornoza@ua.es (E.Z.)

**Keywords:** carbon nanofibers (CNFs), strain sensing, damage sensing, cement composites

## Abstract

Cement composites with nano-additions have been vastly studied for their functional applications, such as strain and damage sensing. The capacity of a carbon nanofiber (CNF) cement paste has already been tested. However, this study is focused on the use of CNF cement composites as sensors in regular concrete samples. Different measuring techniques and humidity conditions of CNF samples were tested to optimize the strain and damage sensing of this material. In the strain sensing tests (for compressive stresses up to 10 MPa), the response depends on the maximum stress applied. The material was more sensitive at higher loads. Furthermore, the actual load time history did not influence the electrical response, and similar curves were obtained for different test configurations. On the other hand, damage sensing tests proved the capability of CNF cement composites to measure the strain level of concrete samples, even for loads close to the material’s strength. Some problems were detected in the strain transmission between sensor and concrete specimens, which will require specific calibration of each sensor one attached to the structure.

## 1. Introduction

The addition of specific particles to confer new properties is usually used to achieve additional functional applications to some materials. For several years, research interest has been focused on these so-called smart materials, i.e., materials capable of responding automatically to certain external stimuli. Among these composites, cement-based materials have played an important role in construction and civil engineering industries. In the particular case of multifunctional cement composites, multifunctionality is achieved by taking advantage of the structural material itself to develop nonstructural functions, without the need of any type of external device. In order to obtain these multifunctional properties, cement materials should be combined with specific conductive admixtures that provide the new composite with a new range of functional applications [1,2,3,4,5,6,7,8,9,10], while mechanical properties are maintained or even improved [11,12,13,14,15,16,17]. Hence, cost would be reduced, as design will be simplified minimizing the use of embedded devices. Functional properties affect different fields of applications, such as anodes for electrochemical chloride extraction [2,3], electromagnetic wave shielding [5], structural monitoring and damage detection [8,10,18], temperature sensors [19], heating and thermal control [20,21,22,23,24,25,26,27], and strain/stress sensors [6,7,8].

The present work is particularly aimed at the use of cement-based composites as strain or damage sensors in concrete elements. The strain sensing capacity of a material is determined by the response to its electrical resistivity in relation to the mechanical stresses applied to it [7]. If a unidirectional compressive stress is applied, the electrical resistance in that direction is proportionally reduced. However, the electrical response would be the contrary if the material was tensioned, i.e., an increase in the resistance will be registered. Both effects are reversible in the material’s elastic range. Hence, electrical changes will be reversible if loads are removed. This application of cement composites is interesting for structural service state monitoring, room occupancy control, or vehicle weighing. On the other hand, a damage sensing mechanism begins at the yielding point, corresponding to the material’s plastic behavior, and can be detected as irreversible changes in the electrical resistivity [28]. 

To achieve these functional applications, the electrical resistivity of regular cement materials should be improved. This can be easily achieved by means of conductive admixtures, such as steel fibers, carbon fibers, graphite powder, graphene, carbon nanofibers (CNFs), or nanotubes (CNTs). Percolation can be defined as the situation where the conductive fibers or particles, randomly dispersed, create a continuous path along the material, which is related to low resistivity values [29]. The minimum admixture dosage that creates these conductive pathways is known as the percolation threshold, as reported by several authors working with different admixtures. For cement composites, in order to show a reversible and stable strain sensing behavior, a conductive admixture is required. However, percolation is not mandatory, i.e., a high conductivity is not needed for strain sensing applications [30].

The self-sensing properties (strain or damage) of cement composites have been studied for different carbon admixtures: carbon fibers [1,30,31,32,33], CNFs [4,28,34,35], CNTs [36,37,38,39], or graphene [40,41]. The strain sensing response depends on the electrical conductivity of the composite [1,30], its water saturation degree [42], the type of electrical measure [7], or the curing age of the material [31]. Besides all the characterization of the sensing phenomena made using small specimens, there are some studies that implement these cement sensors in full-scale structural elements [8,10,43,44]. In order to implement these multifunctional composites in the structural monitoring system, three different approaches have been used: the whole structure is functionalized (i.e., the structural concrete made sensitive), conductive composites are attached to the surface of the structure, or they are embedded when the structural concrete is poured. Howser et al. [43] tested a structure (column and foundation) made completely in concrete doped with CNFs. In this case, the electrical contacts to monitor the resistivity change were embedded in the center of the column at different heights. On the other hand, other studies have fabricated small sensors that are attached to or embedded in regular structures. For example, reinforced concrete (RC) beams have been monitored with different carbon fiber and CNF cement pastes to control strain levels until failure [8]. Another study has reported the use of a CNT cement paste layer (casted directly on top of a regular RC beam) that was used to assess the dynamic behavior of the structure [10]. Finally, the third possibility (embedded sensors) comprised the fabrication of 30 × 40 × 50 mm³ cement pastes (with carbon black as conductive admixture), which were embedded at different depths of a RC beam [44].

The aim of the present work is to study strain sensing and damage sensing properties on CNF cement pastes, which have already been proven useful for other applications [26,27]. Sensors made in this CNF cement paste were attached to concrete elements, which were loaded only in compression. Therefore, the strain or damage level of the concrete was monitored by the electrical resistance changes in the CNF sensors. Another aim was to address different technical issues, the solutions of which are necessary for the practical implementation of this monitoring system. For example, the electrical resistivity changes related to different water saturation conditions are considered here. Finally, different experimental configurations to monitor the electrical resistivity of each sensor are evaluated.

## 2. Materials and Methods 

### 2.1. Materials and Sample Fabrication

Sensors with dimensions 2.5 × 10 × 1 cm³ were fabricated from Portland cement pastes with 5% (by cement mass) CNF addition. This material has been successfully tested in prior research for other functional applications [26,27]. The materials used were Cement type EN 197-1 CEM I 52.5 R, distilled water, carbon nanofibers type GANF-4 supplied by Antolín-Irausa, Burgos, Spain (S.A.). The water/cement ratio was fixed at 1.0 for all sensors in order to attain good dispersion and workability.

CNFs were previously dispersed in the mix water. A double treatment was applied to guarantee a proper CNF dispersion based on prior work tested in cement composites [28]. First, CNFs were added to water and mechanically stirred in an automatic rotatory mixer for 10 min. Afterwards, an ultrasound treatment was applied to this mix using an ultrasound device, model Hielschier UP200S (Teltow, Germany), at maximum power for 5 min. Then, all components (CNFs dispersed in water and cement) were poured into an automatic mixer for 5 min.

**Figure 1:**
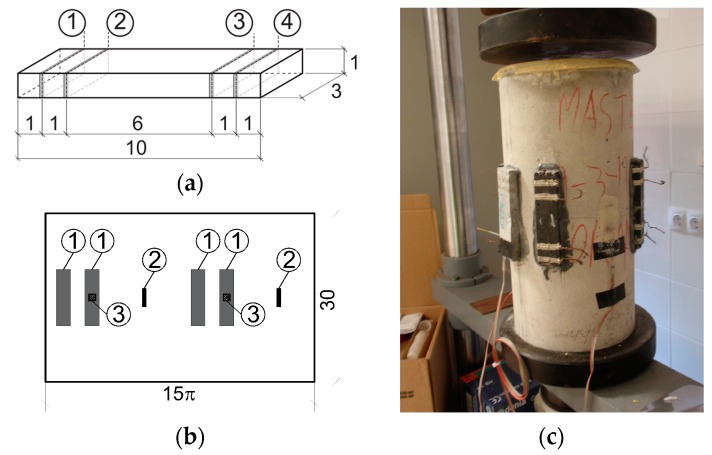
(**a**) Sensor dimensions, in cm, and electrical contact distribution; (**b**) Cylinder unwrapped plane, in cm. 1: sensors; 2: concrete strain gage; 3: sensor strain gage; (**c**) Concrete specimen.

In order to simulate a possible casting method in real structures, instead of being directly poured into the molds, the fresh mix was sprayed using a compressed air pistol (which had been previously tested in other studies [26,45]). Molds were kept in a controlled environment room, 20 °C and >95% relative humidity (RH) for 24 h. Samples were then demolded and conserved at room temperature and water-saturated ambient until 28 days age. Afterwards this curing period, specimens were externally dried, and silver electrically conductive paint (Pelco Conductive Silver 187, Ted Pella Inc., Redding, CA, USA) was applied around the perimeter at four interior planes, which were parallel to the end surfaces. Moreover, four copper wires were wrapped around each silver painted perimeter in order to form four electrical contacts, as needed for the four-probe method (Figure 1a).

Prior to attaching each sensor to a concrete specimen, its volumetric electrical resistivity was analyzed at three different water saturation conditions: First, they were analyzed in water-saturation conditions (100% RH). Specimens were then kept for 1 week inside an electric oven at 50 °C. Finally, they were kept for 28 days under laboratory conditions at 20 °C and 65% RH. For each conservation condition, three different electrical measurements were taken:

- 4 point measurements using only a digital Keithley 2002 multimeter (National Instruments Corp., Austin, TX, USA). Input cables were connected to Electrodes 1 and 4—Figure 1a—and output cables were connected to Electrodes 2 and 3.
- 4 point measurements using a power source (Keithley 6220, National Instruments Corp., Austin, TX, USA) and a multimeter (Keithley 2002, National Instruments Corp., Austin, TX, USA). The power source was used to input a 1 mA fixed current in the external cables and the multimeter was used to register the electrical voltage between the internal cables—Points 2 and 3 in Figure 1a.
- The third method was analogous to the second one, but the current intensity was increased up to 10 mA.

Each resistance (R) value was measured until voltage (V) deviation was lower than 0.1% for a fixed current intensity (I) and resistivity (ρ) can then be calculated using Equation (1), depending on the sample’s section (A) and length (l).

(1) $$R=\frac{V}{I}=\rho \frac{l}{A}.$$

After resistivity tests, sensors were attached to concrete cylindrical specimens (diameter equal to 15 and 30 cm high). Concrete strength and elastic modulus vary between 44 and 50 MPa and between 37 and 43 GPa, respectively. Figure 1b shows the sensor distribution in each concrete sample, while Figure 1c includes an example of one of the specimens. Sensors were attached with an epoxy resin (NURAL27) specific for cement-like materials. The surface of the concrete specimens were pre-treated such that a flat, rough surface was obtained. Strains at both CNF sensors and concrete specimens were measured using HBM strain gages (Darmstadt, Germany) (type 6/120LY41-3-1M for sensors and type 20/120LY41-3-1M for concrete). 

### 2.2. Strain Sensing Tests

**Figure 2:**
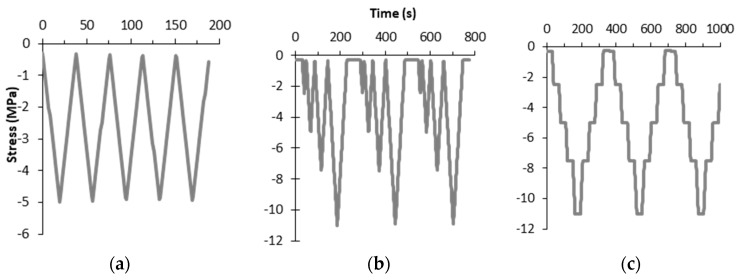
Time history of compressive stress for strain sensing tests: (**a**) standard tests; (**b**) increasing amplitude tests; (**c**) steady stress increments.

Compression loading and unloading cycles were made to concrete specimens monitored with CNF cement sensors. Figure 2 includes the three types of loading time histories used for strain sensing tests. The Strain-1 test comprised consecutive loading–unloading cycles up to a maximum stress level, as shown in Figure 2a, in which five cycles were run. The Strain-2 test, shown in Figure 2b, was defined as progressively higher-stress cycles, which were repeated three consecutive times. Finally, Strain-3 tests, Figure 2c, were steady state cycles, in which the maximum stress was applied in four steps, each of which was maintained for 30 s. Before the load cycles, a 1 mA fixed electrical current was applied to all sensors until steady resistivity values were obtained in order to minimize polarization phenomena. A preload of 5 kN (0.28 MPa) was constantly applied during this calibration phase.

### 2.3. Damage Sensing Tests

**Figure 3:**
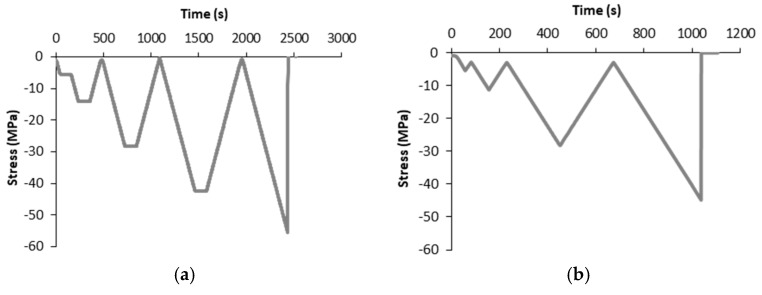
Time history of compressive stress for damage sensing tests: (**a**) steady stress increments; (**b**) increasing amplitude tests.

After all strain sensing tests, damage sensing tests were conducted until each concrete sample failed. Figure 3 shows the two types of damage tests. The Type 1 test, denoted as Damage-1, Figure 3a, was a steady incremental load–unload until failure. The Type 2 test, denoted as Damage-2, Figure 3b, was an incremental load–unload strength test. The loading rate was fixed to 2000 N/s (equivalent to 0.113 MPa/s). The aim of Damage-1 was to analyze the effect of steady loads, while the aim of Damage-2 was to reduce the number of load–unload cycles in order to minimize hysteresis phenomena.

## 3. Results

First, before any sensing test, the electrical resistivity of the sensors was assessed in different saturation conditions. Afterwards, CNF cement sensors were attached to cylindrical concrete specimens, and strain-sensing tests under compression were conducted. Finally, concrete specimens were loaded until failure, while CNF sensors monitored their strain or damage status. The main results of each of these experimental phases are presented and analyzed below.

### 3.1. Electrical Resistivity

Table 1 shows the average values of the electrical resistances monitored (R), the calculated electrical resistivity (ρ), and the relative standard deviation (RSD) for different sensors, different storage methods, and different types of electrical measurements. Method #1 is a four-point measure using only a multimeter; Method #2 is a four-point measure, with a power source at 1 mA and a multimeter; Method #3 is also a four-point measure with a power source at 10 mA and a multimeter. Table 2 includes the average resistivity value for all different measures (i.e., Methods #1–3) made in the same storage conditions. All results showed relatively low deviations (RSD ≤ 4.4%). At every storage condition, the different measurement methods showed minor variations. Nevertheless, as expected, every storage condition showed significant variations in the electrical measurements. The minimum electrical resistance was obtained at 100% RH, and the maximum obtained under laboratory conditions (about three times higher). All results showed a substantially electrically conductive composite. Specimens under 50 °C oven-drying conditions were similar to those dried under laboratory conditions. Though fully saturated samples were the most conductive, measurement dispersion was lower for oven-dried specimens (RSD ≤ 2%) and lowest for those kept in a lab environment (RSD ≤ 1.6%). Moreover, the variation between measuring methods was also lower for non-saturated samples (see Table 2). In any case, the stability of the measurements is highlighted since the samples were monitored every three weeks for 120 days.

According to the previous results, it was decided that the sensors, attached to the concrete specimens, should not be water-saturated. Therefore, they were kept under laboratory conditions, in order to avoid possible damages during oven drying (shrinkage-related cracks). In addition to a lower water concentration in the samples, lower polarization effects were expected when constant DC currents were applied, as the electrolytic contribution to electrical conduction diminishes [46]. Moreover, the high CNF content increased overall conductivity, enhancing electronic conductivity. Finally, to minimize these polarization phenomena, a 1 mA fixed direct current was selected (i.e., the four-point measure of Method #2).

### 3.2. Strain Sensing Tests

**Figure 4:**
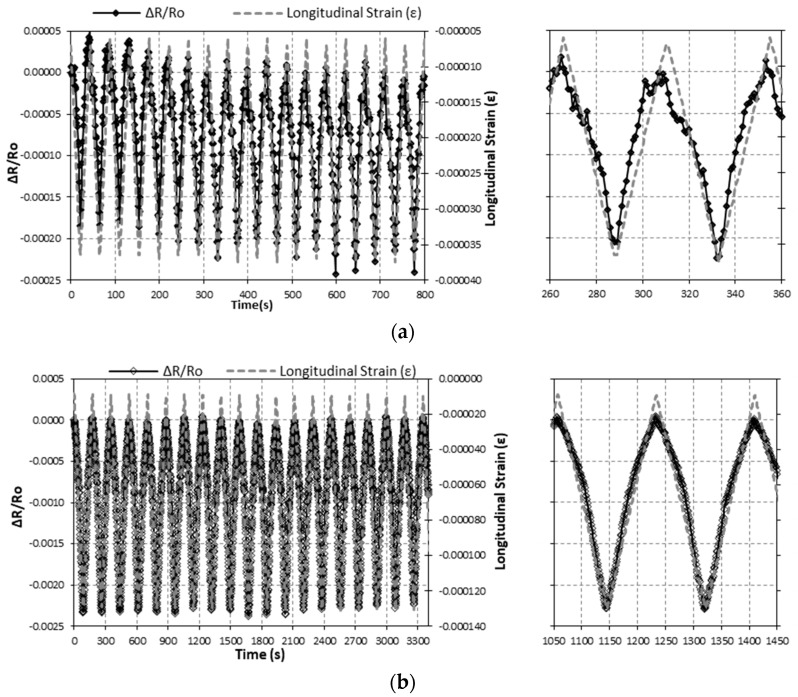
Strain sensing tests results, resistance change, and longitudinal strain curves (7th & 8th cycles zoom on the right) for compression cycles up to (**a**) 22 kN (1.25 MPa) and (**b**) 88 kN (5 MPa).

After resistivity characterization, CNF sensors were attached to concrete specimens, and self-sensing tests were run. Three different test configurations were used, as detailed above in the Materials and Methods section. All of them applied maximum stresses between 1.25 and 5 MPa to guarantee elastic behavior in concrete specimens. Figure 4 shows two Strain-1 type strain test results, in which time history functions registered for both the electrical resistance change of the sensor ($∆R/R_{o}$) and the longitudinal strain (*ε*) of the concrete sample are included. The maximum load applied was 22 kN (1.25 MPa) and 88 kN (5.0 MPa) for Figure 4a,b respectively. The strain-sensing capacity of the sensors (at different loading levels) is confirmed by the similarities between both curves. 

**Figure 5:**
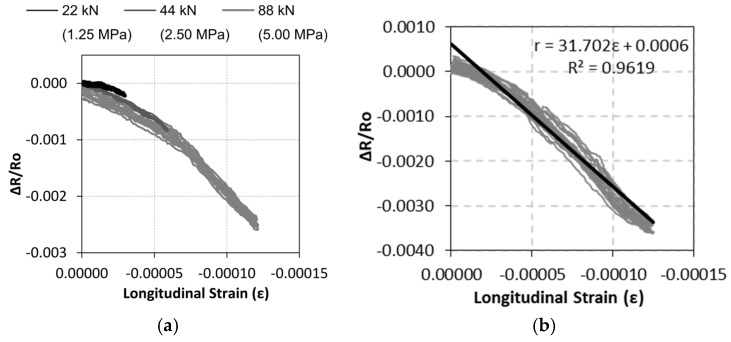
Resistance change vs. longitudinal strain curves for different Sensing-1 type tests (**a**); Curves and linear regression for all measures of the same sensor (**b**).

The results of the sensing tests were plotted as resistance change vs. strain curves (instead of time history functions), and the curves are shown in Figure 5. Each curve in Figure 5a represents different cycles up to a certain stress level (1.25, 2.5, or 5 MPa). Similar trends can be observed regardless of the actual maximum stress. For comparison purposes, the sensitivity of these sensors can be defined by the gage factor (K) as the ratio between the fractional resistance change ($∆R/R_{o}$) and the strain (ε), Equation (2), in which resistance or resistivity ($∆\rho /\rho_{o}$) changes can be considered equivalent, as stated in previous research [1,47]. Figure 5b includes all experimental results obtained by the same sensor after testing under different loading conditions (load rate and maximum stress). Similar behavior can be observed for all testing conditions, as shown by the *R*² coefficient of the linear regression function included. These composites presented good sensitivity, as shown by the gage factor equal to 31.7.

(2) K=∆RRoε≈∆ρρoε.

**Figure 6:**
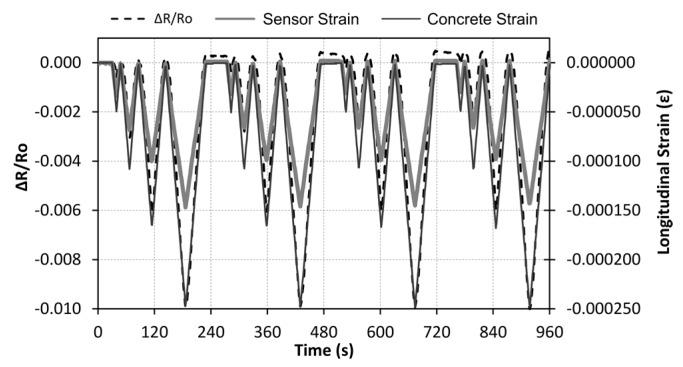
Resistance change and longitudinal strain for the Sensing-2 test.

**Figure 7:**
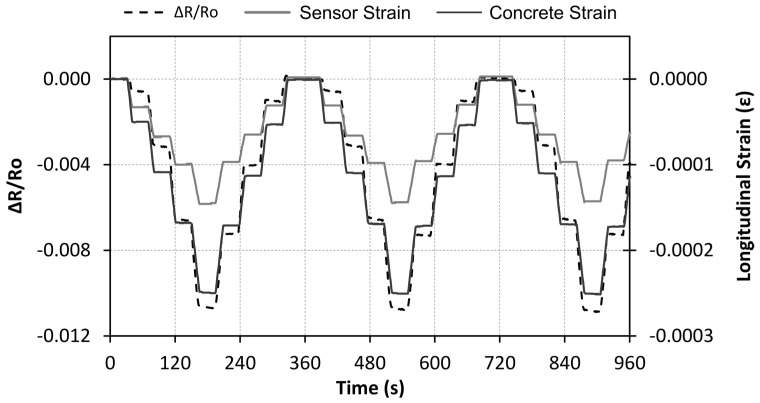
Resistance change and longitudinal strain for the Sensing-3 test.

Similar trends were observed for other types of loading configurations (Sensing-2 and Sensing-3 type tests). Figure 6 includes the strain and resistance change curves for the Sensing-2 tests, in which the amplitude of each cycle is progressively increased between 22 kN (1.25 MPa) and 194 kN (10 MPa). On the other hand, Figure 7 includes the corresponding curves for the Sensing-3 tests, in which maximum stress is achieved after different loading steps. In both cases, similar mechanical and electrical behavior was observed, as in the aforementioned tests with constant amplitudes. However, in these tests, strain gages were also attached to the sensors themselves, showing differences between strain levels on the concrete specimens and CNF sensors. The size of the sensors (1 cm thick and 10 cm long) generate a non-uniform strain distribution. Therefore, sensor strains were 40% lower than the actual strain values of the concrete sample being monitored.

**Figure 8:**
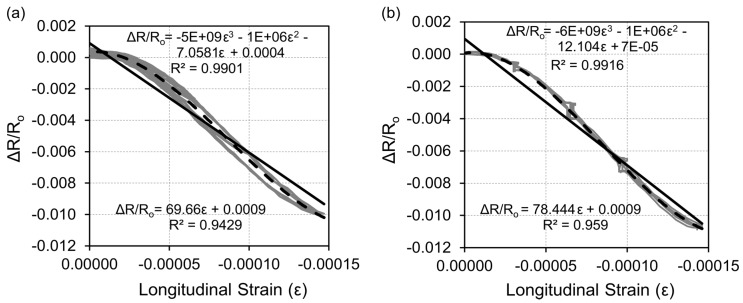
Resistance change vs. strain curves for the (**a**) Sensing-2 and (**b**) Sensing-3 tests.

These results were analyzed in a way similar to that of the initial Sensing-1 tests. The resistance change vs. strain curves are shown in Figure 8. In these cases, good linear regressions were also obtained for both test configurations. Few discontinuities, such as small clusters, were detected for the steady states during the loading step tests (Sensing-3), as shown in Figure 8b. Nonetheless, the gage factors (i.e., sensitivity of the sensor) of both tests were between 69.7 and 78.4. These values were higher than the gage factor in Figure 5, because of the increase in the maximum load, from 5 to 10 MPa. Despite the good *R*² of linear regressions (higher than 0.94), a non-linear behavior can easily be observed in both figures. Therefore, additional regression analysis was made using third degree polynomials, which led to better *R*² coefficients (higher than 0.99). This non-linearity has already been discussed in other work based on a micromechanics approach [48]. 

### 3.3. Damage Sensing Tests

**Figure 9:**
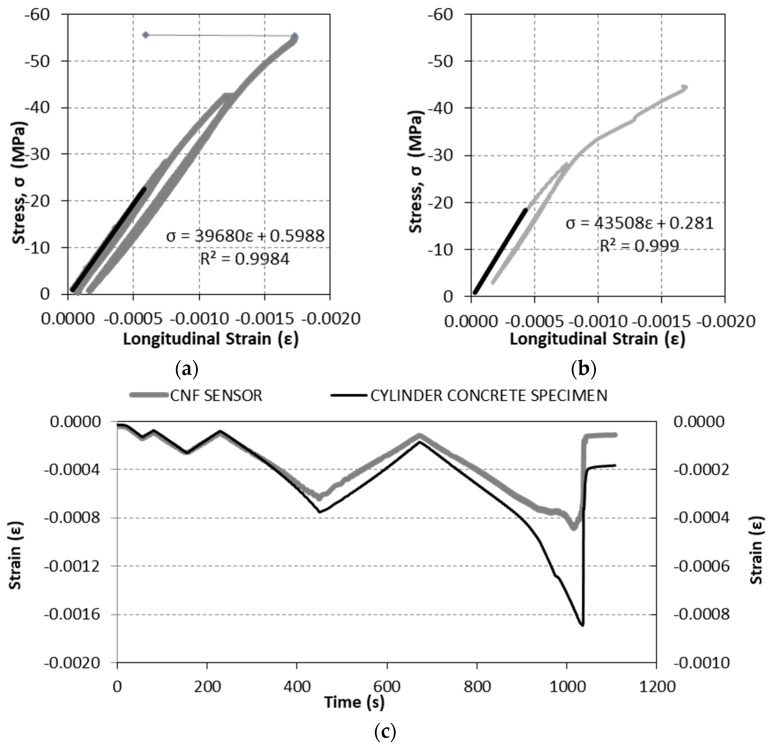
Stress–strain curve for damage sensing tests: (**a**) Damage-1 test; (**b**) Damage-2 test; (**c**) Time history of strains measured in sensors and concrete specimens.

Figure 9 shows the stress–strain curves for two different damage tests, where stress was applied to the concrete specimen in accordance to the setup detailed in the Material and Methods section above. Concrete specimen strain was registered every second during the tests by means of two strain gages adhered to the concrete surface. In black, the elastic range (the first two cycles and part of the third) is denoted, as well as the equation obtained by regression analysis and *R*^2^ value. As expected, after the elastic range, hysteresis can be clearly observed. 

Figure 9c shows the variation over time of both the cylinder concrete specimen (left axis) and the CNF cement paste sensor (right axis). The strain axis ranges have been modified so that the correspondence of both results can be easily observed. As expected, the deformation of the concrete specimen was transmitted to the sensor and as the Young’s modulus is different, both strains are proportional during the elastic range. After surpassing the elastic range, strains do not proportionally match. As observed in the strain sensing tests, the strain of the sensor is approximately 60% of the concrete specimen during the elastic range (first and second cycles and part of the third). After that, this ratio decreases down to around 40%.

**Figure 10:**
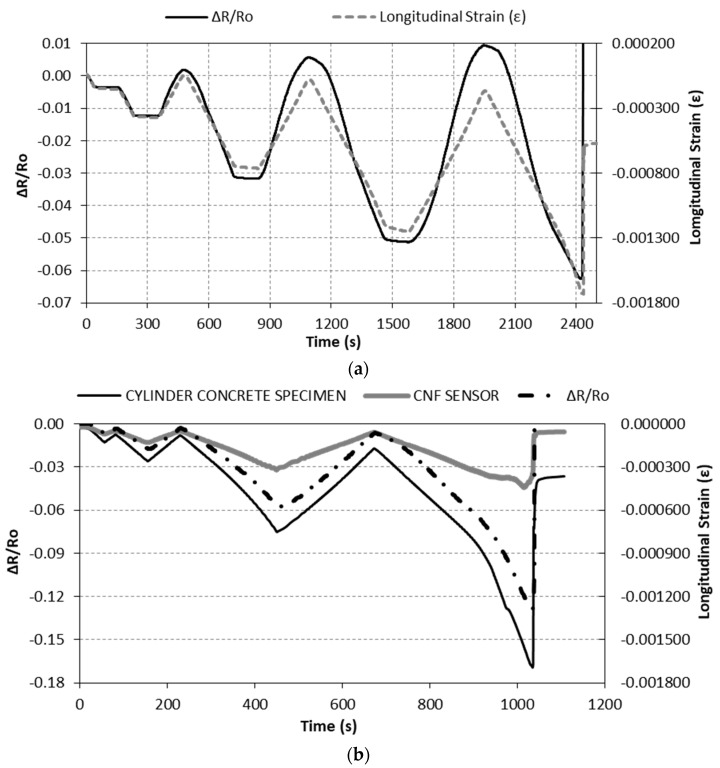
Resistance change and longitudinal strain for (**a**) the Damage-1 test and (**b**) the Damage-2 test.

Figure 10a shows the variation over time of the electrical resistance change of the CNF sensor ($∆R/R_{o}$) (left axis) and the cylinder concrete specimen longitudinal strain (right axis) for the Damage-1 type (see Figure 3) damage sensing test (without a strain gage on the CNF sensor). The strain of the specimen was measured by two strain gages adhered to the surface of the concrete. The electrical resistance change is proportional to the CNF sensor during the elastic range of the test, including the steady steps. After the elastic range is surpassed, the electrical resistance change is no longer proportional to strain. In the figure, this change is represented by the transformation of the electrical response from straight lines to curved lines.

Figure 10b shows the variation over time of the electrical resistance change ($∆R/R_{o}$) and the CNF sensor strain, as well as the longitudinal strain of the cylinder concrete specimen, for a Damage-2 type damage sensing test (with a strain gage on the CNF sensor). The electrical resistance change is proportional to strains measured at both the CNF sensor and the concrete specimen, all over the elastic range of the test, i.e., during the first 300 s approximately. The apex of the third cycle, approximately 450 s, corresponds to a stress of about 28 MPa, i.e., slightly more than 60% of the ultimate stress (44.8 MPa), which means that the concrete specimen is in the plastic phase. The transformation of straight lines into curved lines for all three in the figure ($∆R/R_{o}$, sensor strain, and concrete strain) denotes this change.

## 4. Discussion

Based on analysis of the aforementioned results, strain and damage sensing phenomena and their possible applications, as a consequence of the experimental data, are discussed below.

### 4.1. Strain Sensing Tests

Strain sensing sensitivity of CNF cement pastes has been previously studied in standard samples (4 × 4 × 16 cm³) [28]. The main results of this work highlighted an influence of the maximum load in the composite’s sensitivity to its own strain. Gage factors were measured in the range between 15 and 50 depending on the CNF dosage and the maximum stress applied. This effect has also been observed in the CNF sensors used in the current work. The resistance change–strain curves in Figure 8 presented lower slope values (i.e., lower gage factor, hence lower sensitivity) if strains were lower than 25 µε. Both functions became steeper for increasing strain values, and gage factors consequently increased. Therefore, the gage factor differences between results in Figure 8 (stress up to 10 MPa) and Figure 2 (stress lower than 5 MPa) can be explained by this stress effect on the sensing capability of these composites. These results point to a proper performance of the sensors, as their general behavior matches previously detected trends in CNF paste specimens axially loaded [28].

However, for a real application of these sensors, some issues should first be addressed. For example, strain differences between the monitored element and the sensor are indicated in Figure 6 and Figure 7. Therefore, a particular calibration of each sensor for the service structure should be made prior to putting the system in motion. Nevertheless, the aforementioned results present good linear regressions despite the exact gage factor value, which will depend on relative sensor–structure proportions and geometries or bonding properties. The possibility of embedding the sensors may be an alternative for the real application of these sensors in concrete elements. This technique may solve the strain transmission problems and guarantee more stable humidity conditions. Thus, the resistivity changes due to humidity changes in CNF pastes (as shown in Table 1) can be controlled.

### 4.2. Damage Sensing Tests

After the feasibility of 5% NFC cement paste as sensors capable of sensing the deformation of structural elements (cylinder concrete specimen with dimension of 15 cm× 30 cm, placed with its axis in a vertical plane), the capability of sensing structural damage was analyzed as sensors capable of perceiving structural damage (by simple compression loads).

The variation in the electric resistance change of the sensor is proportional, in the elastic range, to the strain of the sensor and to the strain of the concrete specimen. Similar to other studies [28,30], after the elastic range was overpassed, the electric resistance change ($∆R/R_{o}$) changed from a straight line to a curve, whose curvature increases as stress increases. This phenomenon was also observed in [28], where a strain up to 0.008 at the moment of rupture was observed, whereas in our study of adhered sensors, rupture strain was around 0.000400. Obviously, the Young’s modulus of the sensor is lower than that of the concrete specimen. This behavior is important as it could be used to determine, not only the shift from the elastic range to the plastic range but also the proximity to rupture. 

In any case, due to this strain difference between sensor and structure, CNF sensors did not show great changes in their resistivity, as have other CNF [28] or carbon fiber [7,30] cement composites at higher strain levels. Therefore, these attached sensors can practically measure the strain of the support directly, even for states close to the material’s failure, even though no damage sensing mechanism is triggered [28].

## 5. Conclusions

CNF cement pastes were fabricated as strain sensors and attached to concrete specimens. After all experimental measurements and a discussion of the results, the following conclusions can be drawn:

1. 5% CNF cement pastes are feasible for use as sensors attached to structures subjected to uniaxial compression loads, capable of sensing the strain of the structure, in the elastic range.
2. Such sensors should be previously calibrated due to problems of strain transmission to the concrete structure.
3. Strain sensing is viable from a strain of about 40 µε, which means that these sensors are especially promising for real applications.
4. The loss of linearity between electrical resistance change ($∆R/R_{o}$) and strain (*ε*) of the sensors studied might indicate the limit between the elastic range and the plastic range of the structures to which they adhere. Moreover, as strains in sensors are smaller than the strain of the concrete specimen, CNF sensors might measure the strains of the support even if it is actually damaged.
5. Regression analyses using third degree polynomials, compared to the linear gage factor, result in a more accurate approach to the strain sensing of cement pastes. However, a more complex physical interpretation is required.

## Figures and Tables

**Table 1:** Resistance (average ± RSD relative standard deviation) and average resistivity of 5% carbon nanofiber (CNF) (by cement mass) cement sensors measured under different electrical conditions.

Method	$R_{100\%RH}$ Ω	$\rho_{100\%RH}$ Ω·cm	$R_{50{}^{\circ}C}$ Ω	$\rho_{50{}^{\circ}C}$ Ω·cm	$R_{lab}$ Ω	$\rho_{lab}$ Ω·cm
#1 ^1^	654.5 ± 4.4%	344.5	1760.5 ± 2.0%	926.7	1904.0 ± 1.6%	1002.3
#2 ^2^	696.3 ± 2.9%	366.5	1765.2 ± 2.0%	929.2	1933.6 ± 1.3%	1017.9
#3 ^3^	687.0 ± 2.7%	361.6	1760.9 ± 1.9%	926.9	1920.8 ± 1.3%	1011.1

^1^ Multimeter only. ^2^ External power supply at 1 mA. ^3^ External power supply at 10 mA.

**Table 2:** Resistivity (average ± RSD) of 5% CNF (by cement mass) cement sensors measured under different conditions.

$\rho_{100\%RH}\;$(Ω·cm)	$\rho_{50{}^{\circ}C}$ (Ω·cm)	$\rho_{lab}$ (Ω·cm)
357.5 ± 3.2%	927.6 ± 0.1%	1010.4 ± 0.8%
